# Optimization of injection dose in ^18^F-FDG PET/CT based on the 2020 national diagnostic reference levels for nuclear medicine in Japan

**DOI:** 10.1007/s12149-021-01656-x

**Published:** 2021-07-21

**Authors:** Hiroaki Sagara, Kazumasa Inoue, Hideki Yaku, Amon Ohsawa, Takashi Someya, Kaori Yanagisawa, Shuhei Ohashi, Rikuta Ishigaki, Masashi Wakabayashi, Yoshihisa Muramatsu, Hirofumi Fujii

**Affiliations:** 1 Department of Radiologic Technology, National Cancer Center Hospital East, 6-5-1 Kashiwanoha, Kashiwa, 277-8577 Japan; 2 Department of Radiological Sciences, Graduate School of Human Health Sciences, Tokyo Metropolitan University, 7-2-10 Arakawa‑ku, Tokyo, 116‑8551 Japan; 3 RYUKYU ISG Co., Ltd, 3-78-4 Nantan, Kyoto, 622-0041 Japan; 4 Optical Information Engineering, Systems Innovation Engineering, Graduate School of Advanced Technology and Science, Tokushima University, 2-1 Minamijyousanjima-cho, Tokushima, 770-8506 Japan; 5 Department of Radiation Technology, Faculty of Medical Science, Kyoto College of Medical Science, 1-2 Nantan, Kyoto, 622‑0041 Japan; 6 Clinical Research Support Office, National Cancer Center Hospital East, 6-5-1 Kashiwanoha, Kashiwa, 277-8577 Japan; 7 Division of Functional Imaging, Exploratory Oncology Research and Clinical Trial Center, National Cancer Center, 6-5-1 Kashiwanoha, Kashiwa, 277-8577 Japan

**Keywords:** PET/CT, Diagnostic reference levels (DRLs), Injection dose, Image quality

## Abstract

**Objective:**

Recently, the national diagnostic reference levels (DRLs) in Japan were revised as the DRLs 2020, wherein the body weight-based injection dose optimization in positron emission tomography/computed tomography using ^18^F-fluoro-2-deoxy-D-glucose (^18^F-FDG PET/CT) was first proposed. We retrospectively investigated the usefulness of this optimization method in improving image quality and reducing radiation dose.

**Methods:**

A total of 1,231 patients were enrolled in this study. A fixed injection dose of 240 MBq was administered to 624 patients, and a dose adjusted to 3.7 MBq/kg body weight was given to 607 patients. The patients with body weight-based injection doses were further divided according to body weight: group 1 (≤ 49 kg), group 2 (50–59 kg), group 3 (60–69 kg), and group 4 (≥ 70 kg). The effective radiation dose of FDG PET was calculated using the conversion factor of 0.019 mSv/MBq, per the International Commission on Radiological Protection publication 106. Image quality was assessed using noise equivalent count density (NEC_density_), which was calculated by excluding the counts of the brain and bladder. The usefulness of the injection dose optimization in terms of radiation dose and image quality was analyzed.

**Results:**

The body weight-based injection dose optimization significantly decreased the effective dose by 11%, from 4.54 ± 0.1 mSv to 4.05 ± 0.8 mSv (*p* < 0.001). Image quality evaluated by NEC_density_ was also significantly improved by 10%, from 0.39 ± 0.1 to 0.43 ± 0.2 (*p* < 0.001). In no case did NEC_density_ deteriorate when the effective dose was decreased. In group 1, the dose decreased by 32%, while there was no significant deterioration in NEC_density_ (*p* = 0.054). In group 2, the dose decreased by 17%, and the NEC_density_ increased significantly (*p* < 0.01). In group 3, the dose decreased by 3%, and the NEC_density_ increased significantly (*p* < 0.01). In group 4, the dose increased by 14%, but there was no significant change in the NEC_density_ (*p* = 0.766).

**Conclusion:**

Body weight-based FDG injection dose optimization contributed to not only the reduction of effective dose but also the improvement of image quality in patients weighing between 50 and 69 kg.

## Introduction

Positron emission tomography/computed tomography (PET/CT) using ^18^F-fluoro-2-deoxy-D-glucose (^18^F-FDG) is useful for the functional diagnosis of neoplastic lesions, staging of malignant tumors, and detection of metastases and recurrences [1–3]. With the recent increase in the number of PET/CT procedures, it is becoming increasingly important to control the effective patient radiation exposure without deteriorating image quality [4].

Diagnostic reference levels (DRLs) have been proposed in the United States and in European countries as an important tool for optimizing radiation exposure in nuclear medicine procedures [5, 6]. In Japan, the first DRLs were proposed in 2015, and the injection dose for ^18^F-FDG PET/CT tumor evaluation was set to a constant radioactivity of 240 MBq [7]. However, clinical guidelines for FDG PET imaging issued by the Japanese Society of Nuclear Medicine recommended that the injection dose should be optimized by considering factors that affect image quality, such as body weight [8], and an adjusted injection dose based on body weight was proposed in DRLs 2020, the updated DRLs [9]. This optimization of the injection dose can influence the effective radiation exposure to the patient and the staff, but it may also change the number of counts obtained in the PET/CT procedure, potentially introducing new problems regarding PET/CT image quality [10].

In a previous study, the relationship between injection dose and image quality was investigated using the liver signal-to-noise ratio (SNR_liver_) as a quantitative index of image quality [11, 12]. Although SNR_liver_ can be easily calculated, it has been reported that this index depends on the image reconstruction method and arm position during the examination [13]. The noise equivalent count (NEC) is another image quality index that does not depend on the conditions of image reconstruction, although its formula is more complicated than that of SNR_liver_. Noise equivalent count density (NEC_density_) is a modified NEC in which a patient’s body size is considered. NEC_density_ is also independent of the arm position, and a report indicates that this index is highly correlated with visual scores [14]. As a result, NEC_density_ is becoming popular for evaluating image quality.

As the European and American DRLs do not optimize the injection dose based on body weight [5, 6], it would be beneficial to investigate the usefulness of the optimization method that first appeared in the Japanese DRLs 2020. In this study, we investigated the effects on ^18^F-FDG PET/CT radiation dose and image quality evaluated by NEC_density_ when the injection dose is optimized based on body weight.

## Materials and methods

### Patients

Two groups of patients were included in this study: 722 patients underwent PET/CT with a fixed FDG dose of 240 MBq during the four-month period from April 2018 to July 2018, and 671 patients underwent PET/CT with the optimized FDG injection dose of 3.7 MBq/kg body weight during the four-month period from April 2020 to July 2020. The measured injection doses of both groups and their standard deviations are shown in Table 1.

**Table 1:** Characteristics of study subjects before and after dose optimization

	Before	After
	(*n* = 624)	(*n* = 607)
Age	64.9 ± 13.2	66.7 ± 12.5
	(18–89)	(18–90)
Height (m)	1.63 ± 0.09	1.62 ± 0.08
	(1.37–1.87)	(1.35–1.85)
Body weight (kg)	60.3 ± 9.8	58.8 ± 10.7
	(33–87)	(34–88)
BMI (kg/m^2^)	22.6 ± 3.1	22.4 ± 3.3
	(14.7–36.5)	(14.7–36.8)
Injection dose (MBq)*	239.0 ± 6.3	213.2 ± 40.4
	(220.5–261.6)	(118.9–329.5)
Dose/weight (MBq/kg)*	4.08 ± 0.73	3.62 ± 0.08
	(2.70–7.38)	(3.34–4.06)
Blood sugar level (mg/dl)	102.0 ± 14.9	99.3 ± 15.0
	(45–147)	(61–149)
Uptake time (min)	72.1 ± 9.7	63.4 ± 8.2
	(54–102)	(50–115)
Acquisition time (sec)	120	120

BMI, body mass index

Asterisks (*) denote the actually measured values. The means and their standard deviations are displayed

Among these 1,393 patients, 162 were excluded from the analysis: 98 from the former group and 64 from the latter, for the following reasons: inappropriate injection dose, blood glucose levels greater than 150 mg/dL, acquisition time other than 2 min/bed, and uptake time less than 50 min. Finally, a total of 1,231 cases were analyzed retrospectively: 624 cases with a fixed injection dose and 607 cases with an optimized injection dose based on body weight. This study was approved by the Ethical Review Committee of the National Cancer Center (study No. 2019–012). Due to the study's retrospective design, the requirement for informed patient consent was waived.

### Imaging protocol

In this study, we used a Discovery IQ PET/CT scanner (GE Healthcare, Milwaukee, WI, USA). The detector of this scanner comprised Bi_4_Ge_3_O_12_ (BGO) crystals measuring 6.3 × 6.3 × 30 mm. The transaxial field of view (FOV) was 700 mm, the axial FOV was 260 mm, and 79 axial slices were obtained at the one-bed position. The energy window width was 435–650 keV, and the coincidence time window was 9.5 ns. A matrix size of 192 × 192 and a slice thickness of 3.27 mm were acquired, and the slice overlap between beds was 19 slices. Scattering coincidence correction was performed using a three-dimensional model-based scatter estimation (3D-MBSE) method. This random coincidence method is a single method estimated from the count rate of each detector.

PET images were acquired at 2 min per bed in 3D acquisition mode, and the obtained image data were reconstructed using VUE Point HD and Q. Clear (GE Healthcare) [15].

### Calculation of noise equivalent count density (NEC_density_)

According to the cancer FDG PET/CT imaging method guidelines [16], the NEC at each bed position is given by

1 $${{\varvec{N}}{\varvec{E}}{\varvec{C}}}_{{\varvec{i}}}={(1-{\varvec{S}}{\varvec{F}})}^{2}\frac{{\left({{\varvec{P}}}_{{\varvec{i}}}-{{\varvec{R}}}_{{\varvec{i}}}\right)}^{2}}{\left({{\varvec{P}}}_{{\varvec{i}}}-{{\varvec{R}}}_{{\varvec{i}}}\right)+(1+{\varvec{k}}){{\varvec{R}}}_{{\varvec{i}}}}$$

where *NEC*_*i*_ is the NEC at bed position *i*, *SF* is the scatter fraction, *P*_*i*_ is the number of prompt coincidences in bed position *i*, *R*_*i*_ is the number of contingent coincidences at bed position *i*, and *k* is a coefficient based on the correction method for contingent coincidences (1 for delayed coincidence measurements, 0 otherwise).

*SF* is derived from both phantom studies and the actual patient’s test. The scatter fraction phantom (SF_phantom_) was 0.37, based on the National Electrical Manufacturers Association (NEMA) standard [15].

The number of each coincidence was extracted from the DICOM tag.

NEC_density_ was given by

2 $${{\varvec{N}}{\varvec{E}}{\varvec{C}}}_{{\varvec{d}}{\varvec{e}}{\varvec{n}}{\varvec{s}}{\varvec{i}}{\varvec{t}}{\varvec{y}}}=\frac{\sum_{{\varvec{i}}=1}^{{\varvec{n}}}{{\varvec{N}}{\varvec{E}}{\varvec{C}}}_{{\varvec{i}}}}{{{\varvec{V}}}_{{\varvec{p}}{\varvec{a}}{\varvec{t}}{\varvec{i}}{\varvec{e}}{\varvec{n}}{\varvec{t}}}}$$

where *V*_*patient*_ is the body volume of the imaging area, excluding the brain and bladder areas.

### Data analysis

**Fig. 1:**
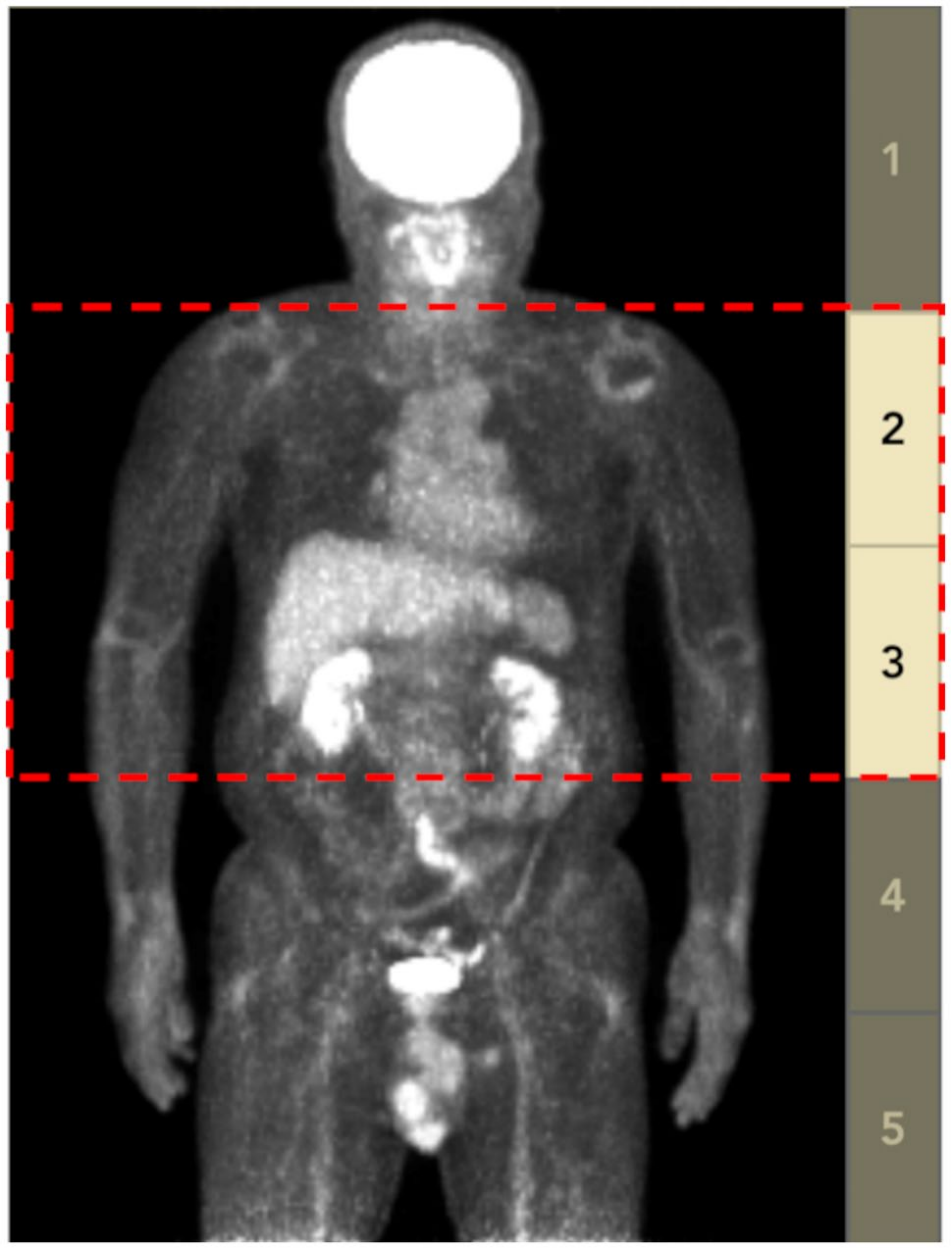
Areas for evaluating a patient’s noise equivalent count. Counts in the area surrounded by dotted lines have been used to calculate the noise equivalent count. The areas, including the brain and bladder have been excluded

PET/CT image data were anonymized and analyzed using a medical image information management system conforming to international standards (onti™, RYUKYU ISG, Kyoto, Japan). NEC and NEC_density_ were calculated using the data extracted from the DICOM tag of PET/CT image data using this information management system (Fig. 1). NEC was fully automatically calculated, and NEC_density_ was automatically calculated by manually choosing the imaging area.

Patients were classified into two groups according to the injection dose: the *fixed-dose group* and the *optimized dose group*. Patient characteristics, such as age, body size, body weight, body mass index (BMI), injection dose, dose to weight ratio (dose/weight), blood glucose level, and uptake time, were compared between the two groups. The effective dose was calculated using the effective dose conversion factor (0.019 mSv/MBq) reported in the International Commission on Radiological Protection Publication 106 [17]. The effective dose and NEC_density_ of the two groups were compared. The two groups were further classified into the following four groups according to body weight: group 1 (≤ 49 kg), group 2 (50–59 kg), group 3 (60–69 kg), and group 4 (≥ 70 kg). Body weight, BMI, dose, dose/weight, effective dose, and NEC_density_ were compared between the four groups.

### Statistical analysis

BellCurve for Excel software (version 3.21, Social Survey Research Information Co., Ltd, Tokyo, Japan) was used for statistical testing. The relationship between body weight, BMI, and logarithm of NEC_density_ was evaluated using Pearson’s correlation coefficient. The injection doses, effective doses, and NEC_density_ were compared between fixed and optimized dose groups using Mann–Whitney *U* test, a non-parametric test, because our data contained some outliers. Statistical significance was set at *p* < 0.05. The effect size, *d*, was calculated as a standardized index independent of sample size [18].

## Results

Characteristics of patients with and without optimization of the injection dose are summarized in Table 1.

**Fig. 2:**
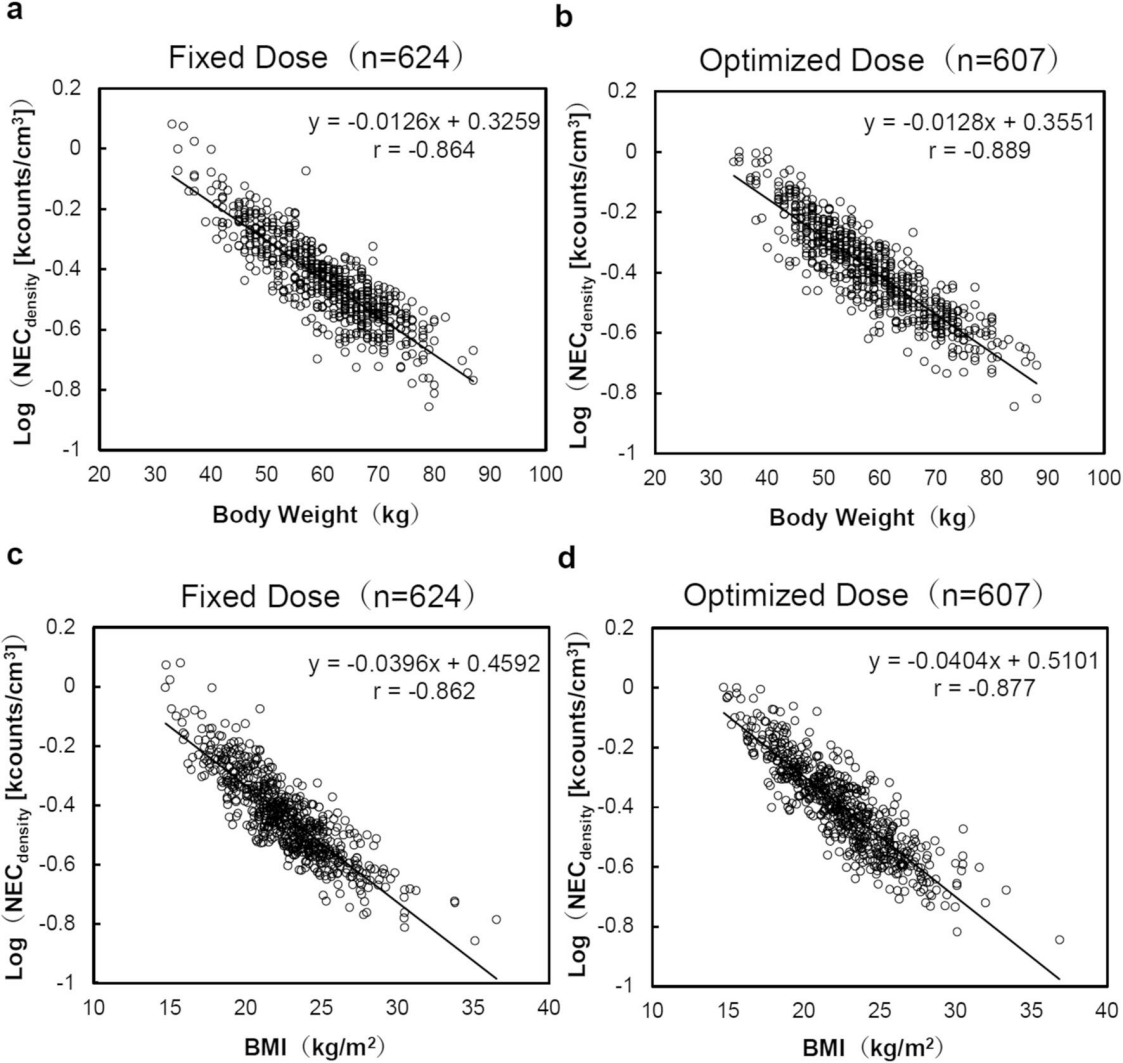
The correlation of the body weight and body mass index with noise equivalent count density. The relationship between the body weight, body mass index (BMI), and the logarithm of noise equivalent count density (NEC_density_) have been evaluated using the Pearson’s correlation coefficient. **a** The correlation between the body weight and the logarithm of NEC_density_, without an optimization of the injection dose; **b** The correlation between the body weight and the logarithm of NEC_density_, with an optimization of the injection dose; **c** The correlation between the BMI and the logarithm of NEC_density_, without an optimization of the injection dose; **d** The correlation between the BMI and the logarithm of NEC_density_, with an optimization of the injection dose

The correlations of body weight and BMI with logarithm of NEC_density_ in both the fixed and optimized injection doses are shown in Fig. 2. When Pearson’s correlation coefficients were calculated, there was a statistically strong negative correlation between body weight and logarithm of NEC_density_ at a fixed dose (*r* = –0.864, *p* < 0.001) and optimized dose (r =  − 0.889, *p* < 0.001). A similar correlation was observed between BMI and logarithm of NEC_density_ at a fixed dose (*r* =  − 0.862, *p* < 0.001) and optimized dose (r =  − 0.877, *p* < 0.001).

**Fig. 3:**
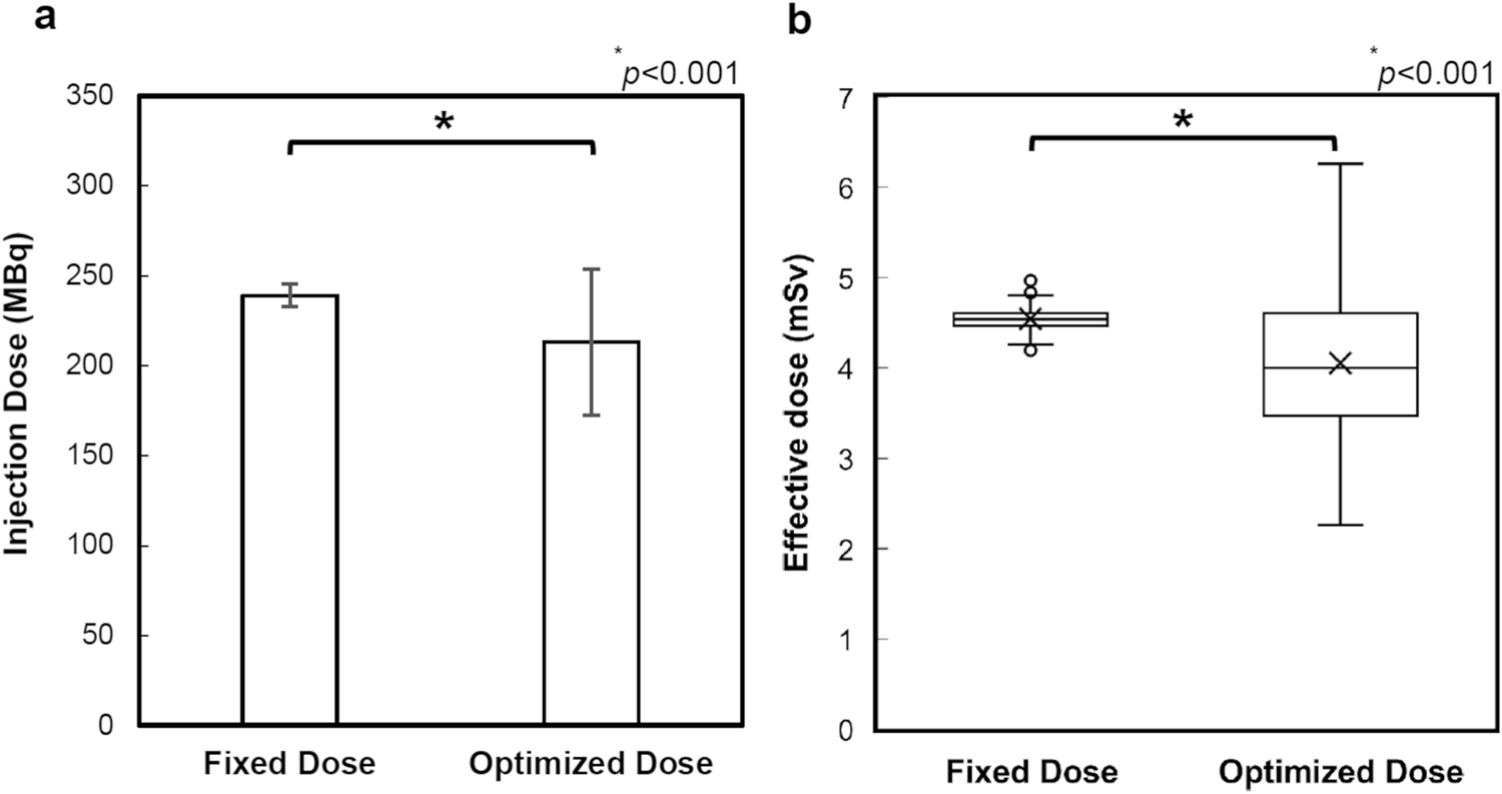
Injection dose and effective dose with and without dose optimization, based on the body weight. The Mann–Whitney *U* test has been performed, and the statistical significance is set at *p* < 0.05. **a** Injection dose; **b** Effective dose

The injection dose and effective dose with and without dose optimization based on body weight are shown in Fig. 3. The optimization of injection dose based on body weight significantly decreased the effective dose by 11%, from 4.54 ± 0.1 to 4.05 ± 0.8 mSv. A statistically significant difference was shown by Mann–Whitney *U* test (*p* < 0.001). Image quality evaluated by NEC_density_ was significantly improved by 10%, from 0.39 ± 0.1 to 0.43 ± 0.2 (*p* < 0.001).

NEC_density_ was larger than 0.2, which is the reference value recommended by the Japanese guidelines [16], in 97.4% of patients with a fixed dose (608 of 624 cases) and in 98.7% of patients with an optimized dose (599 of 607 cases). There was no case in which the NEC_density_ was 0.2 or less with the optimized injection dose. Dose optimization based on body weight reduced the exposure dose without deterioration of image quality in 74.0% (450 of 607) of the patients. The effect size, independent of the sample size, was large for radiation exposure reduction (*d* = 0.90) and small for image quality improvement (*d* = 0.27).

**Fig. 4:**
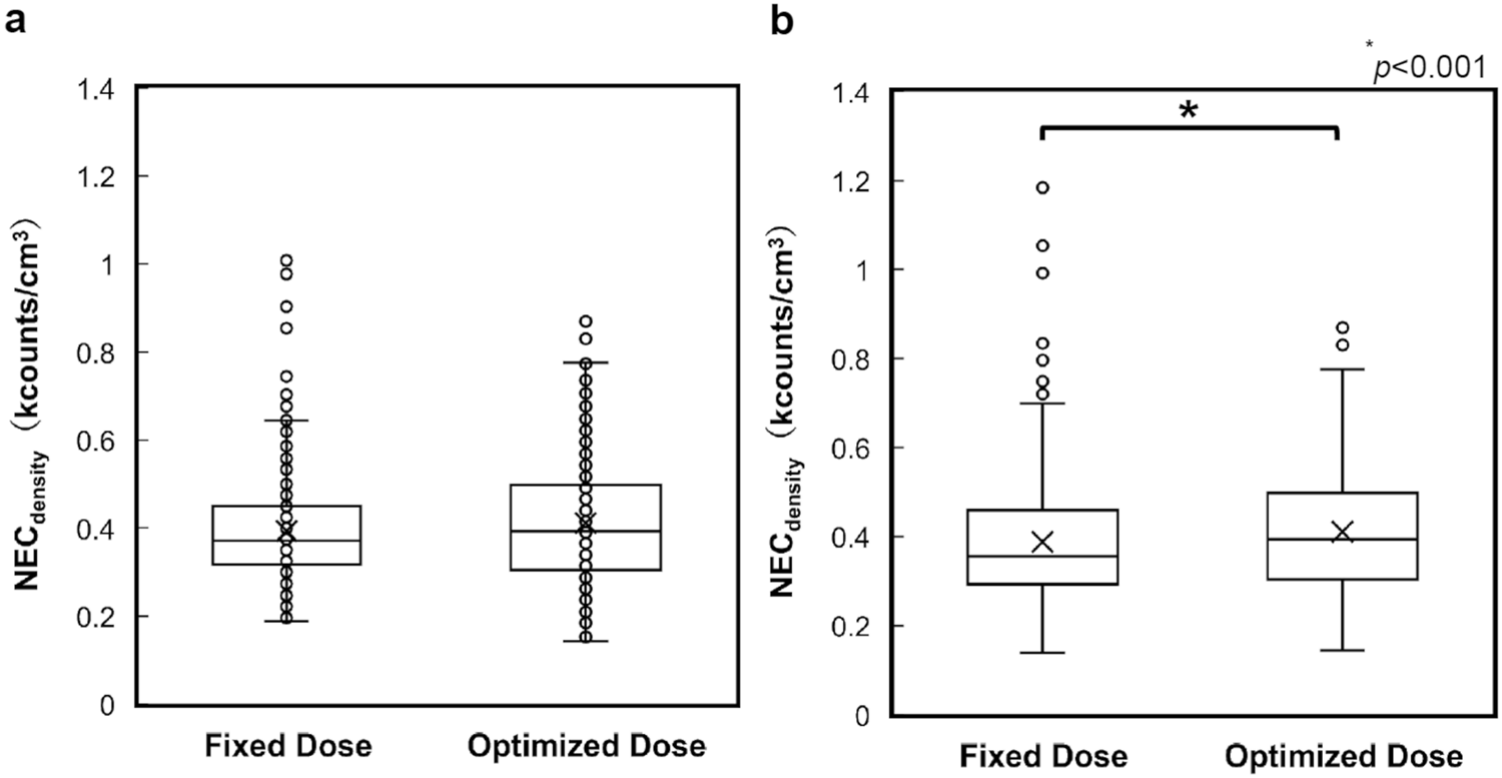
Noise equivalent count density calculated from the SF_phantom_ and SF_patient_, with and without dose adjustment. The Mann–Whitney *U* test has been conducted, and statistical significance is set at *p* < 0.05. **a** NEC_density_ calculated by the SF_phantom_ (*p* = 0.267); **b** NEC_density_ calculated by the SF_patient_ (*p* < 0.001)

The NEC_density_ values calculated using the SF_phantom_ and SF_patient_ are shown in Fig. 4. The NEC_density_ values in fixed and optimized doses were 0.39 ± 0.11 and 0.40 ± 0.11, respectively, when SF_phantom_ was used. They were 0.39 ± 0.14 and 0.43 ± 0.16, respectively, when SF_patient_ was used. NEC_density_ showed no statistically significant difference by Mann–Whitney *U* test when SF_phantom_ was used (*p* = 0.267). However, NEC_density_ was significantly improved after optimization of the injection dose when SF_patient_ was used (*p* < 0.001).

Table 2 shows patient characteristics in the four body weight groups. As for each group, the patients’ mean body weight and BMI were not significantly different with and without dose optimization. As for the injection dose, a statistically significant decrease was shown with optimization in groups 1, 2, and 3 by Mann–Whitney *U* test (*p* < 0.001). However, a statistically significant increase was shown in group 4 (*p* < 0.001).

**Table 2:** Characteristics of study subjects in Groups 1 to 4 before and after dose optimization

Group 1 (≤ 49 kg)	Before	After	Statistical analysis
	(*n* = 100)	(*n* = 130)	*p*-value
Body weight (kg)	44.8 ± 3.9	45.0 ± 3.6	NS
	(33–49)	(34–49)	
BMI (kg/m^2^)	18.9 ± 1.9	18.7 ± 2.0	NS
	(14.7–24.8)	(14.7–23.8)	
Injection dose (MBq)	239.3 ± 7.0	160.9 ± 13.5	< 0.001
	(223.5–252.3)	(118.9–182.3)	
Dose/weight (MBq/kg)	5.36 ± 0.54	3.58 ± 0.07	< 0.001
	(4.61–7.38)	(3.41–4.00)	
Effective dose(mSv)	4.52 ± 0.1	3.06 ± 0.3	< 0.001
	(4.25–4.79)	(3.06–3.47)	

Group 2 (50–59 kg)	Before	After	Statistical analysis
	(*n* = 174)	(*n *= 208)	*p*-value
Body weight (kg)	55.0 ± 2.8	54.5 ± 2.9	NS
	(50–59)	(50–59)	
BMI (kg/m^2^)	21.2 ± 2.0	21.4 ± 2.1	NS
	(16.4–27.2)	(16.7–27.6)	
Injection dose (MBq)	238.8 ± 5.9	197.7 ± 12.0	< 0.001
	(220.5–260.6)	(174.1–235.3)	
Dose/weight (MBq/kg)	4.35 ± 0.25	3.62 ± 0.08	< 0.001
	(3.88–5.11)	(3.34–4.06)	
Effective dose(mSv)	4.54 ± 0.1	3.76 ± 0.2	< 0.001
	(4.19–4.95)	(3.31–4.47)	

Group 3 (60–69 kg)	Before	After	Statistical analysis
	(n = 240)	(n = 148)	*p*-value
Body weight (kg)	64.4 ± 2.9	63.9 ± 2.7	NS
	(60–69)	(60–69)	
BMI (kg/m^2^)	23.6 ± 2.0	23.9 ± 2.0	NS
	(18.9–29.8)	(18.9–30.5)	
Injection dose (MBq)	239.3 ± 7.0	232.2 ± 10.5	< 0.001
	(222.5–261.6)	(212.6–259.1)	
Dose / weight (MBq/kg)	3.73 ± 0.19	3.64 ± 0.07	< 0.001
	(3.29–4.16)	(3.39–3.93)	
Effective dose (mSv)	4.55 ± 0.1	4.41 ± 0.2	< 0.001
	(4.23–4.97)	(4.04–4.92)	

Group 4 (≥ 70 kg)	Before	After	Statistical analysis
	(*n* = 110)	(*n* = 121)	*p*-value
Body weight (kg)	74.1 ± 3.9	74.6 ± 4.4	NS
	(70–87)	(70–88)	
BMI (kg/m^2^)	26.0 ± 2.7	26.3 ± 2.4	NS
	(20.9–36.5)	(22.3–36.8)	
Injection dose (MBq)	239.6 ± 6.0	272.5 ± 17.6	< 0.001
	(223.5–261.3)	(242.0–329.5)	
Dose/weight (MBq/kg)	3.24 ± 0.17	3.65 ± 0.06	< 0.001
	(2.70–3.60)	(3.43–3.80)	
Effective dose (mSv)	4.55 ± 0.1	5.18 ± 0.3	< 0.001
	(4.25–4.96)	(4.60–6.26)	

All subjects have been divided into four groups based on their body weight

BMI, body mass index; NS, not statistically significant (*p* < 0.05)

**Fig. 5:**
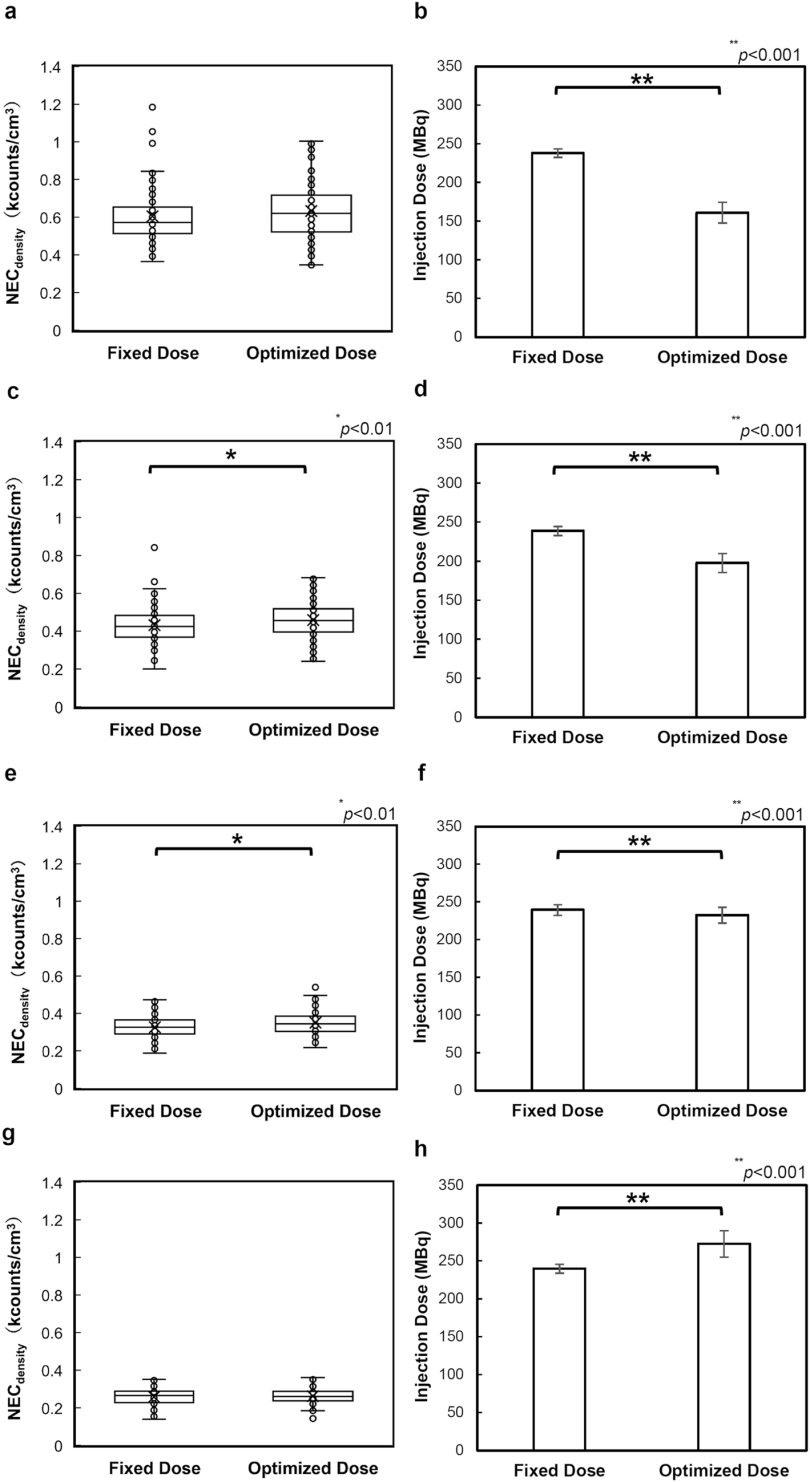
Noise equivalent count density and injection dose, with and without injection dose adjustment in each group. The Mann–Whitney *U* test has been performed, and the statistical significance is set at *p* < 0.05. **a** Group 1 (≤ 49 kg) noise equivalent count density (NEC_density_) (*p* = 0.054); **b** Group 1 (≤ 49 kg) injection dose (*p* < 0.001); **c** Group 2 (50–59 kg) NEC_density_ (*p* < 0.01); **d** Group 2 (50–59 kg) injection dose (*p* < 0.001); **e** Group 3 (60–69 kg) NEC_density_ (*p* < 0.01); **f** Group 3 (60–69 kg) injection dose (*p* < 0.001); **g** Group 4 (≥ 70 kg) NEC_density_ (*p* = 0.693); and **h** Group 4 (≥ 70 kg) injection dose (*p* < 0.001)

The effects of optimization on the NEC_density_ and injection dose in each group are shown in Fig. 5. In group 1, there was no significant difference in NEC_density_ by Mann–Whitney *U* test (*p* = 0.054), although the dose decreased by 32% with optimization. In group 2, NEC_density_ increased significantly (*p* < 0.01), although the dose decreased by 17%. In group 3, NEC_density_ increased significantly (*p* < 0.01), although the dose decreased by 3%. In group 4, NEC_density_ was not significantly improved (*p* = 0.693), although the dose increased by 13%.

## Discussion

The reduction of exposure dose is a critical issue in the field of diagnostic radiology, including nuclear medicine. For this purpose, the concept of DRLs has been utilized in the United States and Europe since the 1990s. Their DRLs propose the 75th percentile dose based on data compiled from facilities in each country or area. In nuclear medicine, the radioactivity of injected radiopharmaceuticals is used as a reference instead of the exposure dose [5, 6]. The movement to optimize the radiation dose in diagnostic imaging in Japan has lagged behind those of Western countries. In Japan, Japan Network for Research and Information on Medical Exposure (J-RIME) published DRLs as late as 2015 and proposed 240 MBq as the recommended injection dose for oncological ^18^F-FDG PET tests [7]. A Discovery IQ PET/CT scanner, which was used in this study, was installed in our institute in 2016, and the injection dose of FDG was fixed to 240 MBq at that time according to DRLs 2015.

However, there is insufficient scientific evidence regarding the usefulness of this fixed injection dose. Another problem is that, when a fixed dose is administered, the PET image quality varies according to body weight or BMI [10, 19]. Therefore, we installed onti™ software, a medical image information management system conforming to international standards, in June 2018. After the ethical review committee of our institute approved this retrospective study in June 2019, we started to investigate the optimization of the injection dose of FDG based on patients’ body weight to reduce the radiation exposure dose, as shown in the clinical guideline for FDG PET, PET/CT 2010 [8]. We set the injection dose of FDG at 3.7 MBq/kg according to Japanese guideline for the oncology FDG PET/CT data acquisition protocol: synopsis of version 2.0 [16].

In April 2020, we started injecting the optimized dose of FDG to all patients after optimizing the injection dose based on their body weight according to the updated Japanese Medical Care Act enacted in April 2020.

That is why we enrolled patients who received FDG PET/CT tests between April 2020 and July 2020 in the group whose injection dose of FDG was optimized according to body weight and those who received FDG PET/CT tests between April 2018 and July 2018 in the group whose injection dose was fixed to 240 MBq.

The usefulness of this optimization was evaluated based on the radiation dose and image quality using the effective dose and NEC_density_, respectively.

There was a strong negative correlation of body weight and BMI with NEC_density_. This means that when evaluated by NEC_density_, image quality deteriorated with an increase in body weight and BMI. Watson et al. [20] reported similar results. However, when the patients were classified into four groups according to body weight, NEC_density_ increased, which means that image quality improved, although the injection dose decreased in groups 2 and 3 (50–69 kg). Therefore, dose optimization based on body weight, which first appeared in DRLs 2020, contributes not only to the reduction of effective dose but also to the improvement of image quality for patients weighing between 50 and 69 kg. However, in group 4 (≥ 70 kg), the injection dose increased by 14%, but the NEC_density_ was not significantly improved. Although the counts of true coincidence are proportional to the injection dose, the counts of random coincidence, which induce noise, increase proportionally to the square of the injection dose. This is why NEC_density_ was not significantly improved in group 4 [21]. Similar results were reported by Nagaki et al., who indicated that deterioration of image quality occurred in subjects weighing over 75 kg [22]. The optimization of injection dose based on body weight to achieve both a reduction in the effective dose and an improvement in image quality is not useful for patients weighing over 70 kg.

In general, when NEC_density_ is calculated, a fixed SF value obtained from a phantom study is usually used. However, it is not suitable to apply the SF obtained from the phantom to the calculation of the NEC_density_ of each patient. In this study, we compared the NEC_density_ obtained using the SF_phantom_ and SF_patient_ with and without dose optimization. When SF_phantom_ was used, the NEC_density_ was not improved by optimizing the injection dose. However, the usefulness of dose optimization to improve NEC_density_ was demonstrated when SF_patient_ was used.

When SF_phantom_ is used for the calculation of NEC_density_, the NEC_density_ is likely to be underestimated in the low-weight group and overestimated in the high-weight group. Therefore, SF_patient_ should be used to calculate the NEC_density_. These findings were suggested by Hosokawa et al., who expected the discrepancy between SF obtained using phantom and that obtained from each subject by a Monte Carlo simulation [23].

We investigated the effects of dose optimization on the improvement of image quality in addition to the reduction in radiation dose based on the concept of DRLs 2020. The results indicated that adjusting the dose based on body weight contributed to an 11% radiation dose reduction and a 10% improvement in image quality. Optimization of the injection dose based on body weight reduced the dose in 74% of cases, and there were no cases in which NEC_density_ deteriorated to less than 0.2, which is the lower limit recommended in the guidelines. Therefore, we can say that the optimization of injection dose based on body weight proposed in DRLs 2020 in Japan is useful in terms of image quality.

The actual injection doses for both fixed and optimized groups were distributed with some deviations. The standard deviation of injection doses for the fixed-dose group was 6.3 MBq, 2.6% of the scheduled injection dose of 240 MBq. In contrast, the standard deviation of injection doses for the optimized dose group was 0.08 MBq/kg, 2.2% of the scheduled injection doses per body weight of 3.7 MBq/kg. Since the effective dose and NEC_density_ were calculated using the actual injection dose for each case, the deviations of injection doses would not affect the effective dose and NEC_density_ results.

In this study, we used 3.7 MBq/kg body weight for the optimization of the injection dose. This value is the achievable dose (AD) recommended in the DRLs 2020 in Japan. The AD, which is the 50th percentile, is the target value recommended for facilities that achieved the reference levels in DRLs in the United States [6]. The results of our current study may contribute to the future revision of DRLs in Japan.

The current study has some limitations. The optimization of injection dose was not useful for subjects classified into group 4 in terms of image quality and radiation exposure dose. It is expected that image quality would be improved by extending the acquisition time rather than adjusting the injection dose for patients with high body weight [11]. In addition, we did not examine the effects of dose optimization on the visual evaluation of PET images. The interpretation of images by physicians should be investigated in the future.

## Conclusions

Optimization of injection dose based on body weight was first proposed in DRLs 2020 in Japan. Our retrospective study using NEC_density_ as an index of FDG PET image quality revealed that this dose optimization method can improve image quality and reduce radiation exposure, especially for patients weighing between 50 and 69 kg. In these cases, the optimized injection dose based on body weight would be superior to a fixed dose in FDG PET.

## References

[1] Beyer T, Townsend DW, Brun T, Kinahan PE, Charron M, Roddy R (2000). A combined PET/CT scanner for clinical oncology. J Nucl Med.

[2] Endo K, Oriuchi N, Higuchi T, Iida Y, Hanaoka H, Miyakubo M (2006). PET and PET/CT using ^18^F-FDG in the diagnosis and management of cancer patients. Int J Clin Oncol.

[3] Fletcher JW, Djulbegovic B, Soares HP, Siegel BA, Lowe VJ, Lyman GH (2008). Recommendations on the use of ^18^F-FDG PET in oncology. J Nucl Med.

[4] Murano T, Tateishi U, Iinuma T, Shimada N, Daisaki H, Terauchi T (2010). Evaluation of the risk of radiation exposure from new ^18^FDG PET/CT plans versus conventional X-ray plans in patients with pediatric cancers. Ann Nucl Med.

[5] European Commission. Radiation protection no. 180. Diagnostic reference levels in thirty-six European Countries 2/2. European Union, 2014. https://ec.europa.eu/energy/sites/ener/files/documents/RP180%20part2.pdf. Accessed May 17, 2021.

[6] Alessio AM, Farrell MB, Fahey FH (2015). Role of reference levels in nuclear medicine: a report of the SNMMI Dose Optimization Task Force. J Nucl Med.

[7] Watanabe H, Ishii K, Hosono M, Imabayashi E, Abe K, Inubushi M (2016). Report of a nationwide survey on actual administered radioactivities of radiopharmaceuticals for diagnostic reference levels in Japan. Ann Nucl Med.

[8] Shishido F, Senda M, Ito K, Inoue T, Kumita S, Sasaki M (2010). Clinical guideline for FDG PET, PET/CT 2010 (in Japanese). Kaku Igaku.

[9] Abe K, Hosono M, Igarashi T, Iimori T, Ishiguro M, Ito T (2020). The 2020 national diagnostic reference levels for nuclear medicine in Japan. Ann Nucl Med.

[10] Sánchez-Jurado R, Devis M, Sanz R, Aguilar JE, del Puig CM, Ferrer-Rebolleda J (2014). Whole-body PET/CT studies with lowered ^18^F-FDG doses: the influence of body mass index in dose reduction. J Nucl Med Technol.

[11] Masuda Y, Kondo C, Matsuo Y, Uetani M, Kusakabe K (2009). Comparison of imaging protocols for ^18^F-FDG PET/CT in overweight patients: optimizing scan duration versus administered dose. J Nucl Med.

[12] Everaert H, Vanhove C, Lahoutte T, Muylle K, Caveliers V, Bossuyt A (2003). Optimal dose of ^18^F-FDG required for whole-body PET using an LSO PET camera. Eur J Nucl Med Mol Imaging.

[13] Shimada N, Daisaki H, Murano T, Terauchi T, Shinohara H, Moriyama N (2011). Optimization of the scan time is based on the physical index in FDG-PET/CT (in Japanese with English abstract). Nihon Hoshasen Gijutsu Gakkai Zasshi.

[14] Mizuta T, Senda M, Okamura T, Kitamura K, Inaoka Y, Takahashi M (2009). NEC density and liver ROI S/N ratio for image quality control of whole-body FDG-PET scans: comparison with visual assessment. Mol Imaging Biol.

[15] Jha AK, Mithun S, Puranik AD, Purandare NC, Shah S, Agrawal A (2019). Performance characteristic evaluation of a bismuth germanate-based high-sensitivity 5-ring discovery image quality positron emission tomography/computed tomography system as per National Electrical Manufacturers Association NU 2–2012. World J Nucl Med.

[16] Fukukita H, Suzuki K, Matsumoto K, Terauchi T, Daisaki H, Ikari Y (2014). Japanese guideline for the oncology FDG-PET/CT data acquisition protocol: synopsis of version 2.0. Ann Nucl Med.

[17] Radiation dose to patients from radiopharmaceuticals. Addendum 3 to ICRP Publication 53. ICRP Publication 106. Ann ICRP. 2008;38(1–2):1–197. 10.1016/j.icrp.2008.08.003 19154964

[18] Cohen J Statistical power analysis for the behavioral sciences, 2nd ed. Mahwah, NJ, Lawrence Erlbaum Associates. 1988;66–7.

[19] Boellaard R, Delgado-Bolton R, Oyen WJ, Giammarile F, Tatsch K, Eschner W, et al. FDG PET/CT: EANM procedure guidelines for tumour imaging: version 2.0. Eur J Nucl Med Mol Imaging. 2015;42(2):328–54. 10.1007/s00259-014-2961-x PMC4315529 25452219

[20] Watson CC, Casey ME, Bendriem B, Carney JP, Townsend DW, Eberl S (2005). Optimizing injected dose in clinical PET by accurately modeling the counting-rate response functions specific to individual patient scans. J Nucl Med.

[21] Halpern BS, Dahlbom M, Auerbach MA, Schiepers C, Fueger BJ, Weber WA (2005). Optimizing imaging protocols for overweight and obese patients: a lutetium orthosilicate PET/CT study. J Nucl Med.

[22] Nagaki A, Onoguchi M, Matsutomo N (2011). Patient weight-based acquisition protocols to optimize ^18^F-FDG PET/CT image quality. J Nucl Med Technol.

[23] Hosokawa S, Inoue K, Kano D, Shimizu F, Koyama K, Nakagami Y (2017). A simulation study for estimating scatter fraction in whole-body ^18^F-FDG PET/CT. Radiol Phys Technol.

